# Management strategies for unanticipated remnants following tooth extraction: A case report

**DOI:** 10.1097/MD.0000000000046801

**Published:** 2025-12-26

**Authors:** Mingyang Liu, Xuewu Zhuo, Li Wang, Chengdong Zhang, Tianqi Zhang, Shengfeng Li, Zhu You

**Affiliations:** a Department of Oral and Maxillofacial Surgery, Shandong Provincial Hospital Affiliated to Shandong First Medical University, Jinan, China; b School of Stomatology, Shandong First Medical University (Shandong Academy of Medical Sciences), Jinan, China; c Department of Stomatology, Zhucheng People’s Hospital, Zhucheng, China; d Department of Health Material Management, Zhucheng People’s Hospital, Zhucheng, China.

**Keywords:** complications, diagnostic imaging, foreign bodies, retained crown fragments, third molar impaction

## Abstract

**Rationale:**

Tooth extraction is a common oral surgical procedure, yet rare complications such as retained tooth fragments may occur. These remnants – including crown fragments, root tips, or other materials – can delay healing, provoke infection, and impair oral function, posing challenges for clinicians. Because fragments may migrate extra-socket and be masked by fibrotic tissue, routine examination can be falsely negative and diagnosis delayed. This case report clarifies when and why early CBCT is warranted to localize retained crown fragments and demonstrates a micro-incision retrieval strategy that minimizes morbidity and supports timely recovery.

**Patient concerns:**

A 27-year-old Chinese female presented with progressive buccal swelling and abnormal sensations 3 months after extraction of the mandibular right third molar (tooth 48). Cone-beam computed tomography (CBCT) identified 2 retained crown fragments.

**Diagnoses:**

Retained crown fragments were diagnosed on CBCT and correlated with the patient’s symptoms.

**Interventions:**

The fragments were surgically removed through a small distobuccal incision adjacent to tooth 47 under local anesthesia, after which symptoms resolved.

**Outcomes:**

Postoperative recovery was uneventful. The patient reported complete resolution of symptoms and improved oral function, with no complications during follow-up.

**Lessons:**

This case highlights the potential for unanticipated remnants after tooth extraction and the need for prompt recognition. Early radiographic assessment – particularly CBCT when indicated – followed by timely surgical removal can prevent long-term complications and support optimal recovery.

## 1. Introduction

Although exodontia (tooth extraction) is a fundamental procedure in oral and maxillofacial surgery, it can lead to various intraoperative and postoperative complications owing to individual anatomical variations, restricted operative fields within the oral cavity, and instrument limitations. Unanticipated remnants (URs) are among the complications that can significantly affect patient recovery after extraction. The size, shape, anatomical location, and proximity to vital structures of these URs can vary. While these URs may remain asymptomatic in the short term, they pose potential risks and can threaten patient health. In particular, the displacement of iatrogenic URs, such as dental instruments or tooth fragments, increases the risk of contamination by the oral microbiota.^[1]^ The removal of URs post-extraction is recommended to prevent recurrent infections, foreign body reactions, and the risk of damage to vital blood vessels and nerve structures. However, unanticipated post-extraction URs are rare. This study explores the causes, outcomes, imaging localization, removal techniques, and prevention strategies, providing clinicians with guidance for managing similar cases.

## 2. Case report

**Figure 1.**
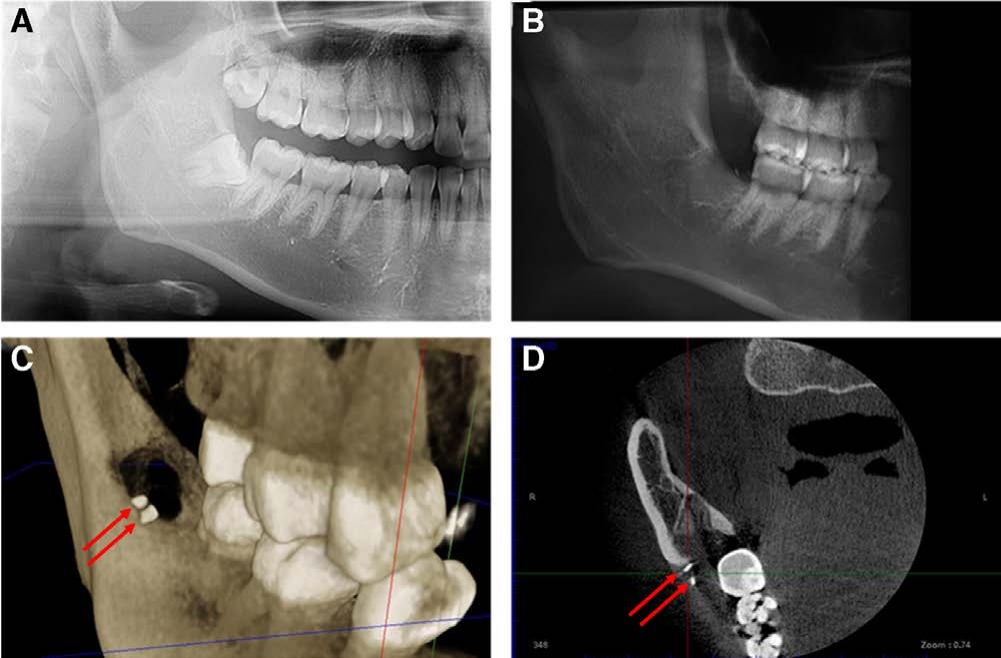
(A) The orthopantomogram of the patient’s impacted tooth on the right mandible before pre-extract; (B) The cone-beam computed tomography after tooth extraction; (C) The reconstructed image after tooth extraction; (D)The horizontal plane of the retained tooth structures.

A 27-year-old Chinese female patient presented with discomfort 3 months after tooth extraction. Panoramic radiographs taken 3 months earlier revealed tooth 18 in a vertical position, while tooth 48 was mesioangularly impacted in a low position, with its root closely associated with the inferior alveolar nerve canal and crown firmly attached to the distal surface of the root of tooth 47 (Fig. 1A). Under local anesthesia, a minimally invasive incision was made on the distal gingiva of tooth 48. A gingival flap operation was performed, and a high-speed handpiece was used to remove the surrounding bone and expose the affected tooth. The tooth crown was sectioned and luxated, followed by root sectioning. The roots were luxated, the dental sac excised, and inflammatory granulation tissue curetted. The socket was irrigated, alveolar bone was reshaped, and the extraction site was sutured following re-disinfection. Sectioning of impacted third molars was performed with a high-speed handpiece under continuous copious irrigation, light pressure, and intermittent cutting to minimize heat generation. Prior studies report no significant difference in postoperative outcomes between high- and low-speed sectioning when adequate cooling is employed, whereas thermal injury is primarily associated with insufficient irrigation and sustained pressure. Our device’s integrated water-spray system maintains temperatures below critical thresholds linked to osteonecrosis, and in this case no clinical or radiographic evidence of thermal damage was observed during follow-up. One week post-extraction, the wound healed well, with intact sutures, which were subsequently removed. The patient did not report any significant discomfort. However, 3 months post-extraction, the patient noticed gradual swelling on the buccal side accompanied by abnormal sensations. Upon examination, the extraction site healed well with no obvious redness or swelling of the surface mucosa. The gingiva on the distobuccal side of tooth 47 appeared hypertrophic compared to that on the contralateral side, felt firm upon palpation, and showed no significant tenderness. The tactile and temperature sensations were normal. Cone-beam computed tomography (CBCT) confirmed the complete extraction of the roots of the right mandibular third molar (Fig. 1B). The reconstructed image (Angle 1) revealed 2 retained tooth structures, appearing as high-density areas, likely representing crown fragments (Fig. 1C and D).

**Figure 2.**
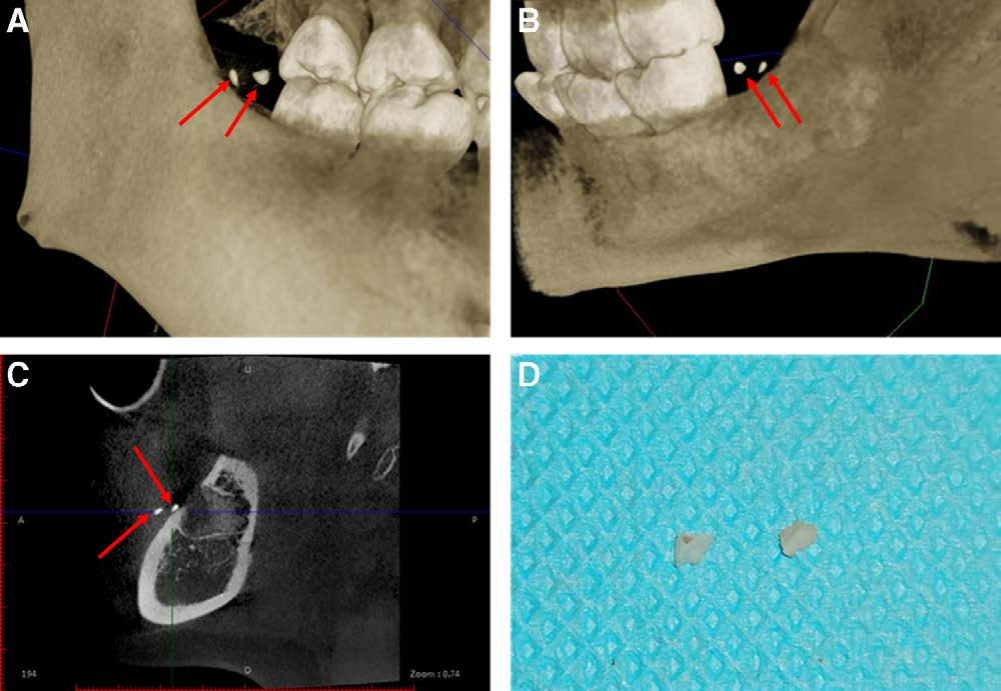
(A) The reconstructed image of the retained tooth structures from the cheek side; (B) The reconstructed image of the retained tooth structures from the tongue side; (C) Another horizontal plane of the retained tooth structures; (D) The photograph of the removed tooth crown fragment.

Further analysis of the CBCT reconstructed images identified a retained tooth crown fragment located outside the extraction socket (Fig. 2A and B). This fragment was located in the distobuccal mucosa of tooth 47 (Fig. 2C). The patient was informed of the condition and provided consent for further management. Following routine sterilization, a distobuccal incision was made on tooth 47 under local anesthesia with articaine hydrochloride and epinephrine tartrate injection, followed by curette probing. No fragment was detected initially; therefore, the scarred area, where the soft-tissue felt firmer, was clamped using a hemostat. The fibrous connective tissue was excised with surgical scissors, revealing an enamel fragment after dissection. Further exploration identified another tooth crown fragment that was successfully extracted (Fig. 2D). Gross inspection confirmed 2 irregular enamel–dentin fragments retrieved from fibrotic submucosa; the socket and adjacent soft-tissues were re-explored with no additional remnants identified. Hemostasis was achieved without complication. Immediate postoperative neurosensory testing (light touch/temperature) over the inferior alveolar and buccal nerve distributions was normal. At 1-week review, the wound had epithelialized and sutures were removed, with marked reduction of swelling and dysesthesia. At subsequent follow-up, the patient reported complete symptom resolution, return to normal diet and mouth opening, and no signs of infection; no adverse events occurred. This study was approved by the Ethical Committee of Shandong Provincial Hospital, affiliated with Shandong First Medical University.

## 3. Discussion

The causes of URs after tooth extraction generally stem from 3 factors: instrumentation, clinician-related issues, and patient-related factors. Retained root fragments are among the most frequently observed URs after tooth extraction, particularly in cases in which the roots are curved, bifurcated, or affected by pathological conditions. Although small root fragments may undergo postoperative resorption, most patients require further intervention. Retained crown fragments are less common but may occur in severe caries or structurally compromised teeth. Residual crown fragments can lead to localized inflammation and infection and potentially compromise the health of adjacent teeth. Fractures of burs and elevators can result from stress concentration, equipment failure, metal fatigue, and improper use. Moreover, nearly half of the cases of broken dental needles were attributed to sudden, unanticipated patient movements.^[2]^ Inexperienced operators, improper use of elevators, incorrect angulation, or excessive force during extraction can lead to elevator-tip fractures. Often, neither the extracted tooth nor the instruments are properly inspected for integrity both before and after the procedure. Patient complaints during follow-up may be overlooked, and clinicians may fail to thoroughly examine the extraction site or consider using radiographic imaging such as X-rays or CBCT for further investigation. The primary cause of leaving fractured burs or elevators in the alveolus during extraction is often surgical oversight and lack of thoroughness. These URs typically require radiographic confirmation followed by surgical removal.^[3]^ Accidental displacement of filling materials into the oral cavity is relatively common during dental procedures. This is particularly common in maxillary fillings, where excess or dislodged material often falls into the oral cavity, although it rarely enters the extraction socket. In patients with cysts, windowing procedures performed post-extraction can lead to inadvertent displacement of the filling materials into the socket. Once the filling materials enter the extraction socket, they should be promptly removed to prevent delayed healing and other adverse outcomes.^[4]^ This complication can also occur in cases of facial trauma with concomitant tooth dislocation. In cases of open soft-tissue injuries to the maxillofacial region, URs such as metal and glass fragments are often present in the wound. Glass fragments, sand, and similar materials were small and sharp. In cases of concomitant tooth dislocation, URs can easily enter the socket. These URs may be overlooked during debridement, necessitating a second surgery for removal, which may increase patient distress. Additionally, maxillofacial trauma can introduce or conceal URs. A large series of mandibular fractures (n = 664) showed high-energy mechanisms (predominantly road traffic accidents), frequent open wounds, and complex repairs (intraoral/extraoral, ORIF), all of which increase the risk of retained foreign material/missed fragments, underscoring the need for early imaging, meticulous debridement, and structured follow-up to avoid late URs.^[5]^ Pediatric mandibular trauma requires growth-conscious management that favors minimal manipulation and conservative fixation (e.g., circummandibular or transosseous wiring) with timely intervention after edema subsides. Because unerupted tooth buds and greenstick patterns make plain films harder to interpret, there is an increased risk of missed/retained fragments; thus, child-appropriate imaging (panoramic/AP when feasible, with adjunct CBCT or ultrasound), meticulous exploration, and layered closure are essential to prevent secondary deformity and late URs.^[6]^ Although these URs are not related to tooth extraction, there is also a risk of URs entering the alveolar socket, as mentioned here. The extraction of horizontally impacted mandibular third molars often necessitates procedures such as gingival flap surgery, bone removal, and crown sectioning. These procedures frequently cause varying degrees of trauma to the surrounding tissues. Postoperatively, injured tissues may exhibit exudation and edema, leading to limited mouth opening, facial swelling, and pain, making wound drainage essential. Both domestic and international researchers have employed various drainage methods, including rubber strips, drainage tubes, and gauze, all of which have demonstrated positive results. The iodoform-Vaseline drain, in comparison, is easy to use and provides sustained drainage due to its prolonged retention time. It effectively reduces and absorbs wound exudates, helping maintain a dry wound environment. In addition, it covers the wound, making it a suitable choice for drainage. Furthermore, iodoform has a mild anesthetic effect that helps alleviate postoperative pain. During follow-up treatment, these drainage materials were removed. Failure to remove a gossypiboma (foreign body granuloma) can lead to delayed healing or foreign body reactions.^[7]^ Beyond passive drains and retained dressings, instrumentation in the mandibular retromolar area itself can create a direct pathway for iatrogenic displacement into deep neck spaces. A case report documented a pain-triggered migration of a retromolar orthodontic mini-screw into the lateral (para)pharyngeal space; CT enabled 3D localization near major vessels, and intraoperative C-arm fluoroscopy guided a minimally invasive intraoral retrieval.^[8]^ This underscores strict prevention during retromolar procedures and the value of image-guided retrieval when displacement occurs.

In some cases, URs may be gradually expelled or broken down by chronic inflammatory processes. However, this mechanism is often inefficient and may not be applicable to all residual types. If the residuals are small or the internal environment is conducive, they may be gradually expelled, although this process can be prolonged and may cause discomfort. Residuals often trigger local inflammatory reactions that can manifest as pain, redness, swelling, and fever. Inflammation is the body’s natural response to URs; however, if persistent, it can progress to chronic inflammation and infection. Residuals can provide a medium for bacterial growth and increase the risk of local infection. Such infections may lead to abscess formation and damage to the surrounding tissues. Prolonged retention of URs can lead to local fibrosis, where connective tissue forms around the object and isolates it. Fibrosis can impair the function of nearby tissues and can potentially complicate future surgical procedures. Residuals can stimulate excessive growth of the surrounding bone tissue, leading to bone overgrowth, which may require additional surgical intervention for removal. Residuals can also cause local bone resorption, affecting the structure and stability of the alveolar bone in some cases. This issue may affect future dental implant procedures and restorative treatments. If the root is left behind and penetrates the maxillary sinus during maxillary tooth extraction, it can result in sinus infections (sinusitis), nasal congestion, and increased discharge. Residuals located near the nerves during extraction can cause nerve damage, resulting in numbness, tingling, or abnormal sensations. Such damages can be temporary or permanent. Residuals can lead to the formation of fistulas, creating abnormal channels between the oral cavity and skin or other body cavities, which can result in persistent infections and pus discharge. Residuals may contribute to the initiation or worsening of periodontal diseases, leading to gum recession and tooth loosening.

A thorough understanding of the patient’s medical history and effective communication are crucial. In addition to obtaining informed consent, discussions should address the nature of surgery, its associated risks and complications, and the significance of patient cooperation.^[9]^ Initially, it is essential to diagnose URs following tooth extraction. This involves a clinical examination for redness, swelling, or tenderness at the extraction site as well as an assessment of oral hygiene. The patient’s complaints, pain levels, signs of infection, and willingness to remove URs were evaluated. Following adequate preoperative psychological preparation for both the physician and the patient, assess the patient’s overall health to rule out serious underlying conditions or contraindications and develop a comprehensive treatment plan. In the oral and maxillofacial regions, long-standing URs often exhibit surrounding fibrosis, rendering them invisible to the naked eye and difficult to palpate. Accurate localization can only be achieved with imaging assistance, which highlights the importance of imaging technology in such cases. Each imaging technique has advantages and limitations; the most appropriate imaging modality should be selected based on the suspected material and its anatomical location. Moreover, factors such as radiation exposure, cost, availability, and patient-specific constraints (e.g., inability to cooperate or the presence of ferromagnetic implants) must be considered. Basic X-ray imaging is straightforward and cost-effective, offering a high sensitivity for detecting URs and identifying radiopaque materials.^[10,11]^ The most commonly used X-ray examination in oral and maxillofacial surgery is orthopantomogram (OPG). Wood or plastic, with densities similar to those of soft tissue, are challenging to visualize using X-rays.^[12]^ If OPG imaging fails to identify URs, a computerized tomography (CT) scan should be performed for further investigation. Cross-sectional CT images enhance detectability and provide accurate anatomical localization of URs compared with OPG methods. Additionally, 3-dimensional CT datasets can be used in intraoperative navigation systems to assist in surgical excision.^[13]^ CT is readily available and can identify fractures or other associated injuries in a single examination, which is particularly beneficial in patients with multiple injuries. CT efficiently detects radiopaque objects such as metals, stones, and glass and helps visualize radiolucent objects such as plastic, wood, or other organic materials not visible on OPG. For example, broken syringe needles are frequently located in the pterygomandibular space, near the mandibular branch. Over 75% of broken needle cases were confirmed using a combination of panoramic radiography and CT imaging.^[14]^ CT scans and angiography are essential for locating fractured needles within tissues and visualizing related structures, particularly when planning the surgical removal of fragments.^[15,16]^ Repeated palpation of tissue is discouraged, and blind removal of fractured needles is not recommended, as the needle may migrate to deeper tissues during such procedures. Moreover, surgical intervention should only be considered after conducting recent imaging studies. Mandibular movements should be restricted during the extraction. Conservative management should be adopted when surgical risks outweigh the clinical benefits.^[17]^ However, radiolucent materials may remain undetectable because of the surrounding tissues, even on CT scans. Contrast-enhanced CT can highlight these indirect signs and provide additional information regarding vascular damage or active bleeding. This information aids in estimating the severity of the injury and in planning the surgical approach. It is important to note that CBCT and digital volume tomography systems, commonly used by dentists and oral and maxillofacial surgeons, have relatively poor soft-tissue contrast. Therefore, traditional (fan-beam) CT is better suited for the detection of radiopaque objects. In patients with metal implants, metal artifacts can significantly obstruct the detection of URs. Although artifact reduction software can improve image quality, the results must be carefully analyzed because algorithms might inaccurately “remove” metal URs.^[18]^ Therefore, evaluating both original and processed images is crucial when URs are suspected. Variations between preoperative imaging and intraoperative anatomy may arise because of soft-tissue distortion and changes in head position during surgery, which can complicate the procedure. These differences make the removal of small URs from deep soft tissues more complex, particularly considering the potential displacement of objects from the time of injury to surgery. To address these challenges, combining intraoperative CT with surgical navigation can facilitate precise removal of small URs from the maxillofacial region. This method utilizes real-time intraoperative CT to ensure accurate correlation between CT images and intraoperative anatomy, minimize errors, and enhance surgical precision. Three-dimensional localization systems, particularly surgical navigation, provide exceptional technical support for precise and minimally invasive removal of maxillofacial URs. These navigation tools assist surgeons in accurately locating and removing URs, reducing the need for repeated exploration, and shortening the duration of the surgical procedure. The integration of intraoperative CT with navigation systems is particularly beneficial in complex cases involving the removal of small URs from the maxillofacial region, where conventional methods may be inadequate.^[19]^ Compared to X-ray and CT scans, ultrasound (US) imaging is a precise and effective method for the localization and diagnosis of URs. US is not limited by the physical properties of the URs, making it particularly useful for detecting radiolucent objects. Research has shown that US is highly effective in identifying soft tissue URs, localizing them before surgery, and confirming their complete removal afterward.^[20]^ It has been shown to reliably detect materials such as wood and plastic, which may not be visible on X-ray or CT scans. URs typically appear as areas of high echogenicity on US, with reverberation artifacts providing additional clues to their presence, and Doppler imaging further enhances detection.^[21]^ US is especially suited for evaluating superficial tissues, offering superior spatial resolution compared with CT or magnetic resonance imaging (MRI). Using US guidance for removing URs retained in soft tissues after tooth extraction is a valuable alternative surgical method. It is cost-effective, repeatable, and carries a low risk for complications. Reports indicate that intraoperative US successfully removed fish bones from the tongue, even when used as a secondary approach after unsuccessful exploratory surgery.^[22]^ Although not widely used, intraoperative US can be quickly employed by skilled radiologists to locate URs post-extraction, thereby promoting faster recovery by minimizing dissection and trauma.^[2]^ However, US has its limitations, including a longitudinal resolution of approximately 2 mm in typical B-mode devices, which may lead to missed detection of very small glass fragments. Therefore, meticulous examination is required, including thorough multi-angle scanning and gentle handling, to avoid deeper tissue displacement of fragments. Additionally, it is difficult to determine the shape of the URs and detect objects smaller than 1 mm. Despite these challenges, US remains a simple, accurate, cost-effective, and safe method for examining glass URs in soft tissue wounds of the maxillofacial area and for preventing the retention of glass fragments, especially in cases of trauma or root fracture requiring tooth extraction. MRI is rarely used in clinical practice to detect or exclude URs owing to cost, convenience, and safety concerns. The exposure of metal URs to the MRI environment can lead to adverse events and potential harm to the surrounding structures.^[23]^ Ferromagnetic materials (such as iron and steel alloys) are radiopaque; therefore, X-ray or CT imaging is recommended before MRI to exclude metal URs.^[24]^ While the positions of URs post-extraction are usually not deep, MRI should be considered if radiography, CT, and US do not exclude the presence of URs for further diagnosis and treatment. Dynamic Navigation Systems (DNS) offer preoperative planning and intraoperative guidance based on 3-dimensional renderings from imaging studies. DNS tracks and displays the precise location of surgical instruments and URs after tooth extraction, thereby providing real-time surgical guidance.^[25]^ DNS has been effectively employed in cases where tooth or root fragments accidentally migrate into the maxillary sinus or sublingual space.^[26]^ It enables surgeons to accurately locate URs, monitor intraoperative conditions, position surgical instruments in real-time, avoid neurovascular damage, enhance surgical accuracy, and reduce operation time.^[27]^ DNS is particularly indicated when URs are trapped in critical areas that could cause severe complications, or when multiple URs are present. If inaccuracies in localization occur due to mandibular movement or soft tissue deformation, careful surgery is necessary. Three-dimensional printed custom static guides and endoscopes may effectively address the navigation deficiencies in such situations.^[28]^ However, DNS has several drawbacks, including the need for additional infrastructure, high cost, increased overall treatment time, potential system errors affecting spatial relationships between reference points and patient data, possible surgical errors, distraction between the screen and surgical field, and potentially increased radiation exposure. Although DNS improves accuracy, appropriate case selection, patient preparation, and fundamental surgical planning and execution remain crucial. Clinical trials with larger sample sizes and longer follow-up periods are needed to validate these findings and to provide robust evidence. Integrating surgical robots with DNS can automate dental surgeries, enhance precision, and address the limitations of DNS. Newly reported cases of URs following tooth extraction are summarized in Table 1.

**Table 1:** Newly reported cases of URs following tooth extraction.

Author	Nature of foreign body	Imaging methods	Methods of the foreign body extraction
Yan et al (2022)^[3]^	Fragments of elevator	CBCT	Operation
Yao et al (2013)^[4]^	Fillings	OPG	Vacuum suction device
Sigron GR et al (2011)^[7]^	Gossypiboma	OPG	Operation
Seif SA et al (2024)^[8]^	Mini-Screw	OPG, CT, and C-Arm X-ray	Operation
Sandre LB et al (2023)^[13]^	Broken dental needles	OPG, CT, and angiography	Operation or retained

CBCT = cone-beam computed tomography, CT = computed tomography, OPG = orthopantomogram.

In this case, the URs had been in place for an extended period, leading to the surface being covered by a thick layer of fibrous connective tissue, which complicated its localization and removal. To address this, we utilized CBCT for an accurate analysis of the fragment’s position. Under local anesthesia with articaine hydrochloride and epinephrine tartrate injection, a small incision was made on the distal buccal side of tooth 47. The thickened tissue was palpated, clamped with hemostat, and excised using surgical scissors. The enamel fragment was exposed by separating the connective tissue. The search continued until the other tooth structures were extracted. In complex extractions, especially when dealing with crushed teeth or split necrotic pulp, there is a risk of retaining the tooth fragments. These fragments are often small and cannot be detected immediately after surgery. The management of these residual fragments should be based on the location and depth of embedding. Direct removal may be appropriate for superficial fragments with no significant inflammatory response. If the fragment is superficial and easily accessible, standard instruments may be used for removal, strictly adhering to local sterilization and hygiene protocols. Careful handling is crucial for deeply embedded fragments, particularly those near vital structures such as nerves or blood vessels. In such cases, preoperative radiological evaluations are recommended to assess the difficulty and potential risks of removal. Radiological evaluations provide detailed information about the fragment’s relationship with surrounding tissues, helping to prevent potential tissue damage and complications. Therefore, a thorough understanding of these factors and proper preoperative preparation are essential to minimize postoperative complications. The difficulty in managing tooth fragments depends not only on their depth but also on their relationship with the surrounding tissues and the reason for their retention. The procedure is relatively straightforward and can be easily used to extract fragments located on the surface of the gingiva. However, for deeply buried fragments within the alveolar bone or extraction socket, it is crucial to exercise caution to avoid damaging the surrounding structures and prevent severe complications, pain, or bleeding. Clinical examination and radiological diagnosis are crucial for identifying URs after tooth extraction. Various radiological methods have advantages and limitations based on their underlying principles. URs, such as wood, plastic, and glass, can complicate diagnosis, making detection more challenging. Radiopaque URs such as metal, glass, or stone can be effectively visualized using X-ray imaging, which offers a low-radiation alternative to CT. US is useful for the detection of superficial URs. MRI is recommended in cases of uncertainty, such as suspected radiolucent objects, persistent wound healing delays, or the need to exclude ferromagnetic materials. Several studies have evaluated the effectiveness of different imaging modalities for detecting common URs under experimental conditions. However, owing to variations in study designs, the results regarding the most effective imaging modality for specific types of URs differ.^[28]^ Clinicians should select appropriate imaging methods based on their experience; in many cases, a combination of imaging techniques is advisable. Post-surgery, the wound should be irrigated with an antimicrobial agent (e.g., chlorhexidine), and hemostatic materials (e.g., bio-cellulose or collagen sponge) should be applied to manage the bleeding. Patients should avoid irritating foods, maintain good oral hygiene, and regularly use mouthwashes (e.g., fluoride-containing) to reduce the risk of infection. Follow-up appointments at 1 week, 2 weeks, and 1 month are recommended to monitor wound healing and check for potential complications such as infection or poor healing. Wound healing should be evaluated along with any postoperative symptoms such as pain or swelling.

## 4. Conclusions

In conclusion, managing and preventing URs after tooth extraction are crucial for ensuring optimal postoperative recovery. By conducting thorough preoperative assessments, employing precise surgical techniques, and providing appropriate postoperative care, the incidence of retained fragments and their associated complications can be significantly reduced. Prompt and accurate treatment of the retained fragments is essential for maintaining oral health. We hope that this article serves as a valuable resource for dentists and other practitioners, aiding in the enhancement of clinical practices and the improvement of patient outcomes study has some limitations. As this is a single case, the generalizability of our observations is limited; larger case series with longer follow-up are needed to validate these findings. Additionally, the relatively short follow-up period introduces uncertainty regarding long-term outcomes. Future research should aim to gather more extensive experience with the removal of URs after tooth extraction for further analysis.

## Acknowledgments

We thank the patient for allowing the publication of this case report.

## Author contributions

**Conceptualization:** Mingyang Liu, Xuewu Zhuo, Shengfeng Li, Zhu You.

**Data curation:** Mingyang Liu, Xuewu Zhuo, Li Wang, Chengdong Zhang, Tianqi Zhang.

**Formal analysis:** Mingyang Liu, Xuewu Zhuo, Chengdong Zhang, Tianqi Zhang.

**Funding acquisition:** Zhu You.

**Methodology:** Xuewu Zhuo, Li Wang.

**Writing – original draft:** Mingyang Liu, Xuewu Zhuo.

**Writing – review & editing:** Shengfeng Li, Zhu You.
